# Integrating retrieval-augmented generation for enhanced personalized physician recommendations in web-based medical services: model development study

**DOI:** 10.3389/fpubh.2025.1501408

**Published:** 2025-01-29

**Authors:** Yingbin Zheng, Yiwei Yan, Sai Chen, Yunping Cai, Kun Ren, Yishan Liu, Jiaying Zhuang, Min Zhao

**Affiliations:** ^1^Biomedical Big Data Center, The First Affiliated Hospital of Xiamen University, School of Medicine, Xiamen University, Xiamen, China; ^2^Meteorological Disaster Prevention Technology Center, Xiamen Meteorological Bureau, Xiamen, China; ^3^School of Software Engineering, Taiyuan University of Technology, Taiyuan, China

**Keywords:** large language models, mistral, SBERT, triage systems, retrieval-augmented generation-based physician recommendation, RAGPR model

## Abstract

**Background:**

Web-based medical services have significantly improved access to healthcare by enabling remote consultations, streamlining scheduling, and improving access to medical information. However, providing personalized physician recommendations remains a challenge, often relying on manual triage by schedulers, which can be limited by scalability and availability.

**Objective:**

This study aimed to develop and validate a Retrieval-Augmented Generation-Based Physician Recommendation (RAGPR) model for better triage performance.

**Methods:**

This study utilizes a comprehensive dataset consisting of 646,383 consultation records from the Internet Hospital of the First Affiliated Hospital of Xiamen University. The research primarily evaluates the performance of various embedding models, including FastText, SBERT, and OpenAI, for the purposes of clustering and classifying medical condition labels. Additionally, the study assesses the effectiveness of large language models (LLMs) by comparing Mistral, GPT-4o-mini, and GPT-4o. Furthermore, the study includes the participation of three triage staff members who contributed to the evaluation of the efficiency of the RAGPR model through questionnaires.

**Results:**

The results of the study highlight the different performance levels of different models in text embedding tasks. FastText has an *F*_1_-score of 46%, while the SBERT and OpenAI significantly outperform it, achieving *F*_1_-scores of 95 and 96%, respectively. The analysis highlights the effectiveness of LLMs, with GPT-4o achieving the highest *F*_1_-score of 95%, followed by Mistral and GPT-4o-mini with *F*_1_-scores of 94 and 92%, respectively. In addition, the performance ratings for the models are as follows: Mistral with 4.56, GPT-4o-mini with 4.45 and GPT-4o with 4.67. Among these, SBERT and Mistral are identified as the optimal choices due to their balanced performance, cost effectiveness, and ease of implementation.

**Conclusion:**

The RAGPR model can significantly improve the accuracy and personalization of web-based medical services, providing a scalable solution for improving patient-physician matching.

## 1 Introduction

Web-based medical services have significantly enhanced healthcare accessibility by improving convenience and efficiency through features such as remote consultations, streamlined scheduling, and enhanced access to medical information (1). Nevertheless, challenges persist, particularly in delivering personalized physician recommendations (2). The diverse array of medical professionals and the varying needs of patients complicate the effective identification of suitable physicians. Currently, most triage processes depend on manual recommendations made by schedulers to guide patients to the appropriate departments or practitioners (3). The increasing volume of consultations reveals the limitations of such manual methods in maintaining quality and professionalism in healthcare delivery (4). Moreover, the intermittent availability of schedulers can disrupt patient access and continuity of care, underscoring the need for a sophisticated recommendation model.

A substantial number of health-related websites now incorporate symptom checker tools (5) that offer preliminary assessments based on user inputs, employing decision trees (6) or rule-based methodologies (7, 8). Following this initial evaluation, the system proposes possible medical conditions and recommends relevant healthcare providers. Notably, machine learning algorithms, including collaborative filtering (9) and content-based filtering (10), have been investigated for their efficacy in recommending physicians by analyzing patient history, preferences, and demographic data. Advances in technology, particularly in natural language processing, present promising opportunities to utilize extensive datasets for generating tailored and precise recommendations.

The Retrieval-Augmented Generation (RAG) (11) framework presents a promising strategy for enhancing the precision and personalization of medical recommendations. Originally designed for handling fact-based inquiries within conversational models (12), RAG comprises two key components: a retriever (13) that locates relevant documents and a generator (14) that synthesizes these documents into coherent outputs. Embedding models play a crucial role in this process by providing linguistic representations that encapsulate semantic meanings in the form of numerical vectors, which are essential for retrieval systems. Simultaneously, generators, such as OpenAI’s GPT series of large language models (LLMs) (15), have demonstrated significant proficiency in producing human-like text and understanding, influencing numerous natural language processing applications, including automated customer support and content generation. By integrating information retrieval with generative modeling, RAG allows systems to generate contextually rich responses that incorporate relevant external data sources (16), thus grounding responses in factual information and substantially improving accuracy while reducing the likelihood of misleading or erroneous outputs.

This study investigates the potential of applying RAG to improve the accuracy, reliability, and contextual relevance of physician recommendations. The objectives include analyzing the limitations of existing web-based medical services, evaluating the effectiveness of RAG in this context, and developing a framework for its implementation to enhance patient-physician matching. Ultimately, the research aims to provide insights that could markedly advance the personalization and efficacy of web-based medical services, thereby improving patient satisfaction. The study addresses the following research questions: How can RAG be utilized to effectively integrate extensive medical documents to provide personalized healthcare for patients? What impact do large language models and embedding models have on the quality of personalized recommendations? How effective is the RAG system in the context of medical services, particularly concerning the precision, recall, and F_1_-score of recommendations?

## 2 Methods

**Figure 1:**
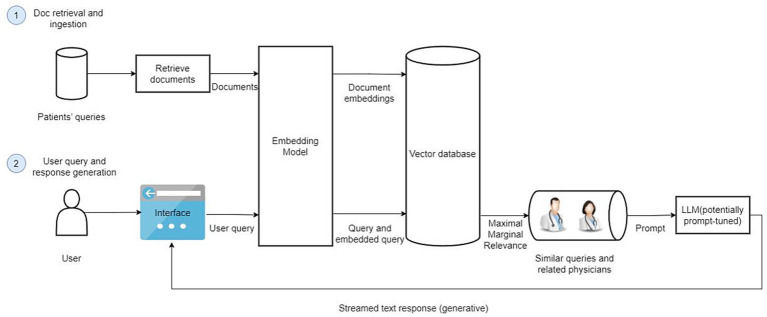
The architecture of RAGPR model. Firstly, a substantial dataset consisting of 646,383 web-based medical documents is curated, anonymized, and formatted for optimized retrieval and analysis. Document embeddings are generated using embedding model, capturing the semantic essence of texts. These embeddings are subsequently stored in a vector database, facilitating efficient similarity searches. Secondly, upon initiation of a user query, the model employs an embedding transformation, replicating the embedding technique used for document processing, ensuring consistency and accuracy. The system retrieves the top six most relevant queries, aligning them with associated physician data. Subsequently, a local large language model is prompted with this contextual information, enabling the generation of comprehensive and contextually recommendations.

The Retrieval-Augmented Generation-Based Physician Recommendation (RAGPR) model, as illustrated in the accompanying figure, comprises two principal components: document retrieval and ingestion, and the generation of user queries and responses. A comprehensive account of the methodology is presented below (Figure 1).

### 2.1 Data collection and preprocessing

The research process commenced with the collation of medical documents pertaining to patients. The training dataset was comprised of 646,383 web-based medical documents, collected from the Internet Hospital of the First Affiliated Hospital of Xiamen University. The documents spanned the years 2016 to 2023. Subsequently, data preprocessing was conducted to ensure that the data were anonymized, structured, and formatted for efficient retrieval and analysis. Each document in this dataset includes the textual query, de-identified codes for the physician and the patient, as well as information on the physician’s department and response time. In contrast, the test dataset consisted of 965 web-based medical documents obtained from Hugging Face, each containing a disease label and a textual query.

### 2.2 Feature extraction and storage

**Figure 2:**
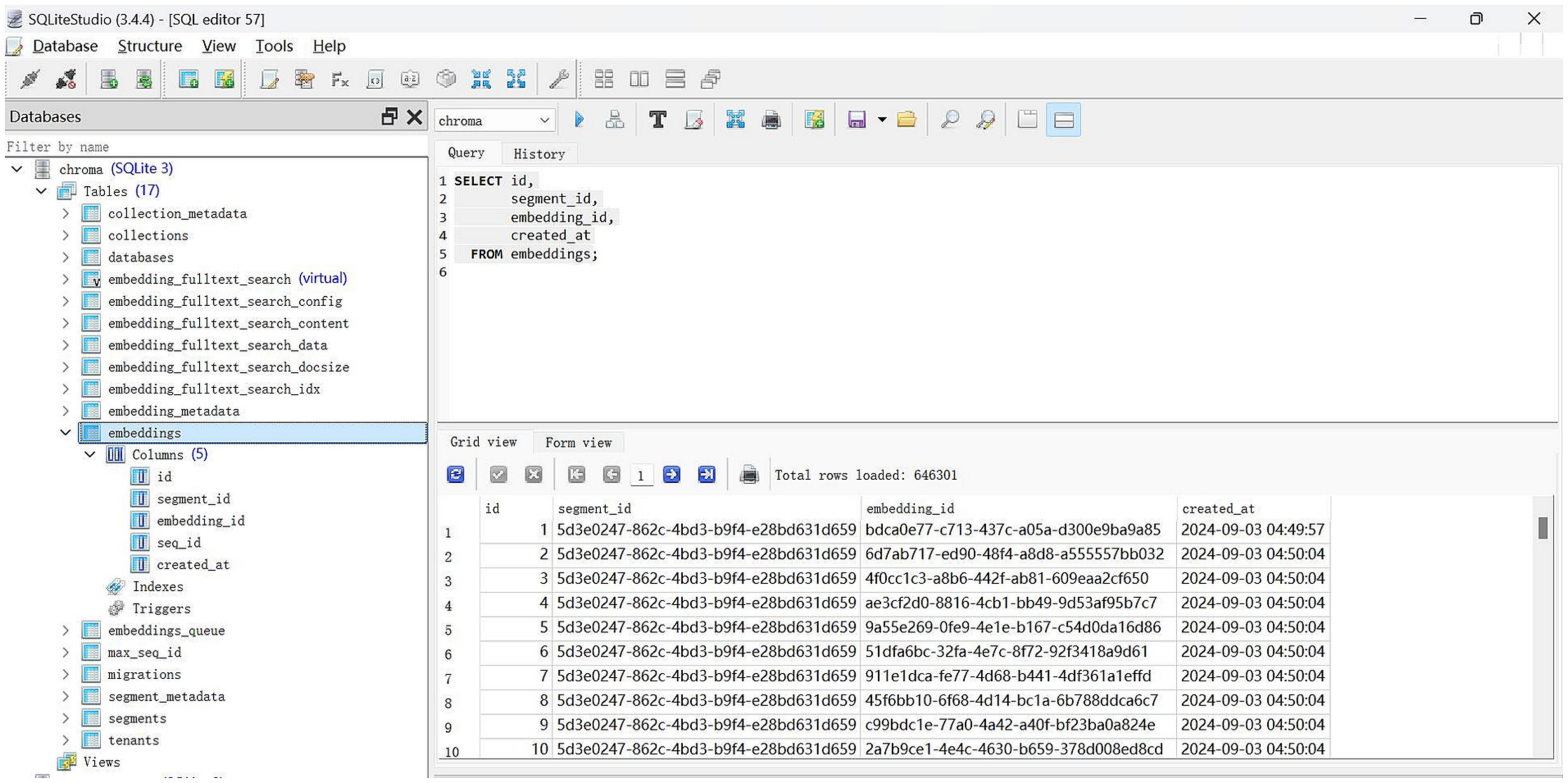
The screenshot of the vector database.

The process of document analysis employs an embedding model that utilizes a pretrained Sentence-BERT (SBERT) model (17), specifically “distilute-base-multilingual-cased, “to transform textual information into numerical embeddings. The resulting embeddings capture the semantic essence of the text (18). The embedding process reduces the dimensionality of the data while preserving semantic similarity, thereby facilitating efficient data storage and retrieval. The resulting document embeddings are stored in Chroma (19), a specialized vector database optimized for managing high-dimensional data. This optimization allows for the rapid execution of similarity searches and serves as a repository for all vector representations of the processed documents, ensuring their accessibility for future retrieval (Figure 2).

### 2.3 User query and response generation

The system’s user interface is developed using the Vue framework (20) and connects to the backend service via a RESTful API (21). It is designed to help users clearly articulate their information needs, allowing the system to efficiently process requests. The user’s input query is then transformed into an embedding using the same embedding model employed in processing the documents. This ensures compatibility and comparability between the document embeddings and the query embedding.

Maximal Marginal Relevance (MMR) is a technique employed in information retrieval to identify documents that are not only pertinent to a given query but also exhibit diversity in relation to those previously selected. The system implements MMR by comparing the embedding of the user query with the embeddings of each stored document. This method effectively reduces redundancy and enhances the coverage of various aspects of the query within the selected documents. Subsequently, the system retrieves the top 6 closely matched queries along with the corresponding physician information for the next step.

A locally constructed LLM was developed using the LLaMA (22) architecture, incorporating the Mistral-7B (23) model for pre-training parameters. This LLM is provided with a prompt that includes similar retrieved queries and related physician information. The prompt facilitates the LLM in generating coherent and contextually appropriate natural language responses. This generative capability ensures that users receive not only straightforward data retrieval but also insightful interpretations and explanations, thereby enhancing their understanding and aiding in the decision-making process. The prompt is as follows: “You are an assistant for question-answering tasks. Use the following pieces of retrieved context to recommend a department and physicians with the shorter response time. The output must be in JSON format and contain only department and physicians.”

### 2.4 Evaluation

The evaluation of the proposed RAGPR model’s effectiveness employed three key metrics: precision, recall, and F_1_-score. Precision is defined as the ratio of correctly identified positive samples to the total number of samples predicted as positive. This metric indicates the accuracy of the model in its positive predictions. In contrast, recall quantifies the proportion of actual positive samples that the model accurately identifies, thus highlighting the model’s ability to detect all pertinent instances. The F_1_-score is a balanced measure that calculates the harmonic mean of precision and recall, providing a comprehensive assessment of the model’s performance. The formulas for precision, recall, and F_1_-score are outlined in Equations 1–3, where TP denotes true positives, FP denotes false positives, and FN denotes false negatives:

(1) $$\mathrm{Precision}=\frac{TP}{TP+FP}$$

(2) $$\mathrm{Recall}=\frac{TP}{TP+FN}$$

(3) $$F1-\mathrm{score}=\frac{2.\left(\mathrm{Precision}\;.\;\mathrm{Recall}\right)}{\mathrm{Precision}+\mathrm{Recall}}$$

### 2.5 Baseline experiments

For the embedding models, FastText (“cc.zh.300”) (24), SBERT (“distiluse-base-multilingual-cased”), and OpenAI’s “text-embedding-3-large” were used to examine their performance on the test dataset, which consisted of 965 web-based medical documents. Each document contained a disease label and a textual query. To facilitate visualization, t-distributed Stochastic Neighbor Embedding (25) was initially applied to reduce the dimensionality of the embeddings. The classification performance of these models was then assessed using precision, recall, and F_1_-score as evaluation metrics.

For the LLMs comparison, GPT-4o, GPT-4o-mini, and Mistral were employed, focusing on their precision, recall, and F_1_-score on the test dataset. Furthermore, to evaluate the rationality of physician recommendations generated by these LLMs, a questionnaire was administered to three staff members involved in triaging. The participants were asked, “Based on your area of expertise, how would you rate the match between physician and the query?” Responses were measured using a 5-point Likert scale (26), with scores ranging from 1 (very inappropriate) to 5 (very appropriate). This evaluation did not involve relabeling the test dataset, but was used to assess whether the model’s predictions were consistent with the professional judgment of these human experts in a triage scenario. The Mann–Whitney *U* test (27) was employed to determine if these assessments revealed any statistically significant differences.

## 3 Results

### 3.1 Data set summary

The dataset consists of 646,383 consultation records involving 193,675 patients and 858 physicians across 44 departments. According to Table 1, male patients constitute 32.95% (*n* = 212,983) of the records, while female patients make up 67.05% (*n* = 433,400). The age group most represented among patients is 20 to 39 years, accounting for 54.6% (*n* = 352,907) of the total consultations. Notably, senior physicians handled the majority of consultations, with 62.65% (*n* = 404,958) attributed to them. Additionally, the majority of response times were recorded at less than 90 min, comprising 38.13% (*n* = 246,472) of the total.

**Table 1:** Summary of the characteristics of the collected data records (*N* = 646,383).

Characteristic		Value, *n* (%)
Gender		
	from Male	212,983 (32.95)
	from Female	433,400 (67.05)
Age of patient at consultation (years)		
	<20	118,484 (18.33)
	20–39	352,907 (54.6)
	40–59	125,957 (19.49)
	>60	49,035 (7.58)
Physicians’ professional title		
	Junior	10,766 (1.67)
	Intermediate	45,892 (7.1)
	Subsenior	184,767 (28.58)
	Senior	404,958 (62.65)
Physicians’ response time (minutes)		
	<90	246,472 (38.13)
	91–180	65,732 (10.17)
	181–270	41,033 (6.35)
	271–360	28,845 (4.46)
	>360	126,943 (19.64)
	Not response	137,358 (21.25)

### 3.2 Evaluating the performance of embedding models

**Figure 3:**
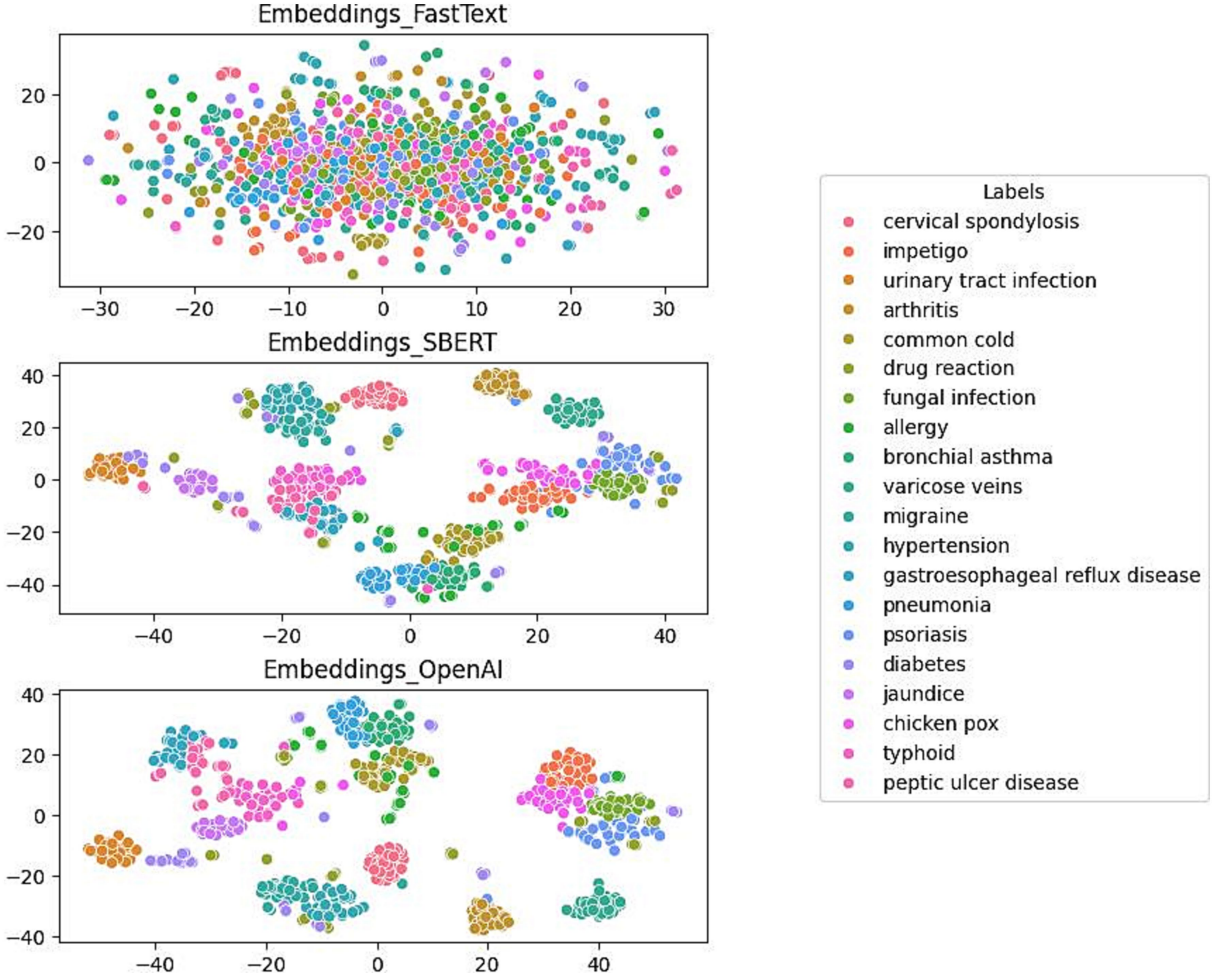
Comparison of FastText (top), SBERT (middle) and OpenAI (bottom) in terms of Clustering.

Figure 3 presents a comparative analysis of the clustering performance of three distinct embedding models using the test dataset. The models FastText, SBERT, and OpenAI were specifically developed for the purpose of labeling medical conditions. The evaluation focuses on the models’ efficacy in differentiating conditions such as cervical spondylosis, urinary tract infection, allergy, and diabetes, which are represented with distinct color-coded labels.

The initial plot illustrates the moderate capacity of the FastText model to differentiate between a numbers of medical conditions. Although the model is successful in creating clusters of similar labels, there is considerable overlap, indicating that there are challenges in effectively separating data points with identical labels. In contrast, the SBERT model demonstrates enhanced clustering capabilities, achieving a more distinct separation among different medical condition labels. This enhancement suggests SBERT’s increased proficiency in distinguishing between conditions. The final plot reveals the performance of the OpenAI model, which exhibits the most distinct clustering. It forms well-defined, tightly grouped clusters corresponding to individual medical conditions and shows minimal overlap between different labels.

Table 2 provides a comparative analysis of the classification performance of three distinct embedding models: FastText, SBERT, and OpenAI, specifically in the context of medical condition labels using the test dataset. The analysis employs a classification model to predict disease labels from the embeddings of disease description texts. For each model, the precision, recall, and *F*_1_-score metrics are presented. The FastText model yielded a precision of 0.52, a recall of 0.44, and an *F*_1_-score of 0.46. In contrast, SBERT and OpenAI exhibited markedly superior performance, with both attaining high precision (0.95 and 0.97, respectively), recall (0.95 and 0.96, respectively), and *F*_1_-scores (0.95 and 0.96, respectively). These findings suggest that SBERT and OpenAI are more efficacious in accurately classifying medical condition labels from textual descriptions than FastText. In light of these findings and additional considerations, such as affordability, data security, and ease of migration, the study ultimately determined that the SBERT was the optimal embedding model.

**Table 2:** Classification performance of FastText, SBERT, and OpenAI.

Embedding models	Precision	Recall	*F*_1_-score
FastText	0.52	0.44	0.46
SBERT	0.95	0.95	0.95
OpenAI	0.97	0.96	0.96

### 3.3 Evaluating the performance of large language models (LLMs)

Table 3 presents a comparative analysis of three LLMs—Mistral, GPT-4o-mini, and GPT-4o—with a focus on their performance in terms of precision, recall, and *F*_1_-score in relation to physician recommendations using the test dataset. The Mistral model demonstrated a precision of 0.95, a recall of 0.94, and an *F*_1_-score of 0.94, indicating a balanced and efficient performance across all metrics. The GPT-4o-mini exhibited a precision of 0.95, which was comparable to that of Mistral. However, it demonstrated a slightly lower recall of 0.90 and consequently a reduced *F*_1_-score of 0.92. In contrast, the GPT-4o model exhibited a slightly lower precision (0.94) but a higher recall (0.97), resulting in the highest *F*_1_-score (0.95) among the models analyzed. Overall, the GPT-4o model demonstrated superior performance in synthesizing precision and recall, as reflected in its *F*_1_-score.

**Table 3:** Comparative analysis of Mistral, GPT-4o-mini and GPT-4o.

LLMs	Precision	Recall	*F*_1_-score
Mistral	0.95	0.94	0.94
GPT-4o-mini	0.95	0.90	0.92
GPT-4o	0.94	0.97	0.95

### 3.4 Rationality evaluation of LLMs

Table 4 presents a Mann–Whitney U test conducted on three pairs of LLMs using the test dataset. The Mistral model has been assigned a rating of 4.56, while the GPT-4o-mini has been rated 4.45 and the GPT-4o has been rated 4.67. The comparison between the Mistral and the GPT-4o-mini yielded a *p*-value of 0.003, indicating a statistically significant difference. The p-value for the comparison between Mistral and GPT-4o is 0.01, indicating a notable difference. Furthermore, the comparison between GPT-4o-mini and GPT-4o yielded a *p*-value of 0.001, thereby affirming the statistical significance of the difference. Considering the study’s findings and additional factors such as affordability, data security, and ease of migration, the research ultimately concluded that the Mistral was the most suitable choice for implementation.

**Table 4:** The Mann–Whitney *U* test conducted on three pairs of LLMs.

Comparison	*p* value
Mistral vs. GPT-4o-mini	0.003
Mistral vs. GPT-4o	0.01
GPT-4o-mini vs. GPT-4o	0.001

### 3.5 Case study

**Figure 4:**
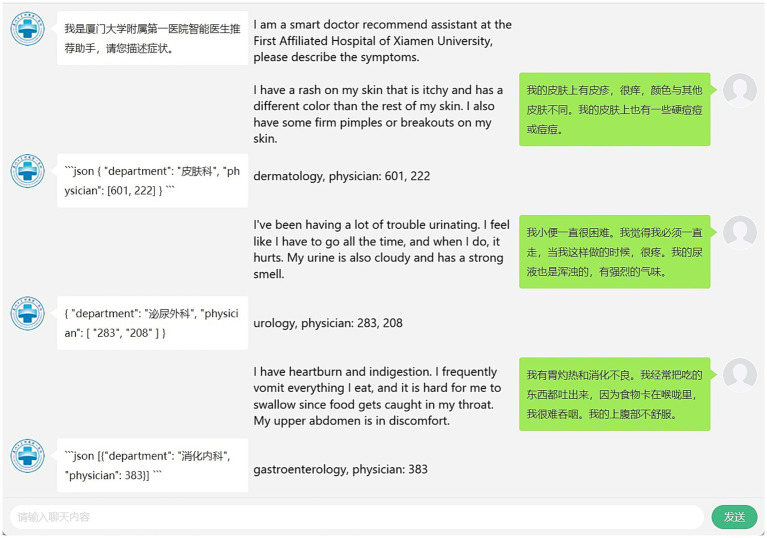
Performance of RAGPR model in mapping symptoms to medical departments and physicians. The layout of the image is divided into three sections: on the right are the human queries, on the left are the responses generated by the model, and in the middle are the interpretations of these interactions.

As illustrated in Figure 4, the RAGPR model demonstrates the capability to accurately identify medical specialties and recommend corresponding medical departments and physicians, based on a randomly selected set of symptom descriptions from the test database. For instance, the model correctly associates dermatological symptoms with the dermatology department and gastrointestinal symptoms with the gastroenterology department. This demonstrates that the RAGPR model effectively processes natural language descriptions to provide relevant medical recommendations, highlighting its potential for application in medical diagnostic systems.

## 4 Discussion

### 4.1 Principal findings

This study introduces an innovative physician triage algorithm called the RAGPR model, designed to enhance the accuracy and efficiency of web-based medical consultations. In our assessment, we evaluated various embedding and large language models to determine the most suitable options based on criteria such as cost-effectiveness, data security, and ease of migration. Consequently, the SBERT and Mistral models were selected as the optimal choices. The RAGPR model demonstrates an improved ability to accurately match patients’ queries with physicians’ specialties.

### 4.2 Reasons behind the performance achieved by the three algorithms

The performance of FastText, SBERT, and OpenAI’s embedding models in the context of medical condition classification is reflective of their respective architectures and capabilities. FastText, although useful for capturing word representations, showed moderate clustering performance with noticeable overlap among medical conditions. This is primarily due to its focus on word-level embeddings without accounting for sentence-level semantics, limiting its effectiveness in distinguishing nuanced medical terms.

SBERT, on the other hand, provided a substantial performance boost. Its architecture, designed to derive sentence-level embeddings, allowed for more refined semantic understanding, resulting in distinct clustering for different medical labels. The ability to capture the contextual meaning of sentences led to significantly higher precision, recall, and F1-scores, making it highly effective for classifying medical conditions based on textual descriptions.

OpenAI’s embedding model exhibited the most pronounced clustering capabilities, indicating its superior understanding and representation of semantic content. Its advanced architecture, likely with larger training datasets and refined algorithms, contributed to tightly grouped clusters and minimal label overlap. However, when considering additional factors such as cost, SBERT was identified as the optimal choice for the application, balancing high performance with practical implementation advantages.

### 4.3 Feasibility and potential extensions of the proposed model

The successful implementation of the RAGPR model in healthcare is contingent upon the existence of a robust IT infrastructure that is capable of handling large volumes of data, facilitating real-time processing, and integrating seamlessly with existing systems such as electronic health records (28). This may require the upgrading of existing systems or the adoption of cloud-based solutions (29) that offer scalability and flexibility. For the model to be widely adopted, it is essential that healthcare professionals receive adequate training to ensure effective use and the development of trust in its capabilities. Such training should include an understanding of how the model generates recommendations and the interpretation of its results. Furthermore, a comprehensive cost–benefit analysis is essential to assess whether the long-term benefits, such as enhanced efficiency, accuracy, and patient outcomes, justify the initial investment. Clearly articulated value propositions, such as reducing diagnostic errors, optimizing physician workload, or improving patient satisfaction, are vital for garnering stakeholder support.

One potential avenue for advancement within the field of healthcare is the integration of the RAGPR model into real-time decision support systems (30). Such systems could provide real-time recommendations during patient consultations, alerting physicians to potential problems such as drug interactions or abnormal test results, and suggesting next steps based on the latest clinical guidelines. This not only increases the efficiency of the visit, but also improves the quality of care by providing timely, evidence-based support. While current models focus on physician recommendations, future research could explore expanding these models to other areas, such as surgical recommendations, chronic disease management, or mental health support. In addition, models could be tailored to specialty areas, such as oncology, cardiology, or pediatrics, to support complex decision-making processes. Another promising extension is the development of personalized medicine frameworks using predictive analytics. By analyzing patient-specific data over time, the model could predict future health risks, recommend preventive measures, and tailor treatment plans to a patient’s unique health profile. This shift from reactive to proactive healthcare could significantly improve long-term patient outcomes.

### 4.4 Real-world application challenges

The implementation of the RAGPR model in healthcare is confronted with a multitude of considerable challenges, one of the most pivotal being the protection of patient data. The utilization of such confidential data for training and deployment must adhere to rigorous privacy regulations, such as HIPAA (31) in the United States and GDPR (32) in the European Union. Breaches of patient confidentiality can have profound legal and ethical ramifications. It is, therefore, of paramount importance to implement robust data encryption, anonymisation techniques, and secure data handling protocols to maintain patient trust and regulatory compliance.

A further significant challenge is the integration of the RAGPR model with existing healthcare IT infrastructure, such as electronic health records systems. This process can be hindered by a number of factors, including compatibility issues, inconsistencies in data format, and concerns regarding interoperability. It is not uncommon for the various systems utilized by healthcare institutions to lack the capacity to interact seamlessly with the model, necessitating substantial customization and development efforts.

Another challenge is the potential for bias, particularly when trained on datasets that are not representative of the population under study. Such bias has the potential to perpetuate or even exacerbate existing disparities in healthcare access and outcomes. For example, if the training datasets predominantly reflect certain demographic groups, the model’s performance for underrepresented populations may be compromised, which could result in unequal treatment. To mitigate these biases, it is essential to employ diverse and representative training datasets and to incorporate fairness constraints during model development. This ensures that healthcare solutions are equitable.

Furthermore, the RAGPR model must undergo continuous adaptation in order to remain relevant and accurate in the context of evolving medical knowledge and practice. This necessitates the implementation of continuous learning frameworks that permit the model to update its knowledge base in response to new medical evidence, guidelines, and emerging diseases. Continuous monitoring, retraining, and validation mechanisms are essential to guarantee that the model provides recommendations that are up-to-date and reliable.

### 4.5 Limitations and future directions

The study has several important limitations. Firstly, the study was conducted exclusively within a single hospital, potentially limiting the applicability of the results to other settings or populations. Secondly, the dataset included irrelevant questions, such as “Doctor, will you be available tomorrow? Where can I find you?” These questions could introduce bias into the analysis. Lastly, a significant limitation of deep neural networks is their opacity, which refers to their lack of transparency in providing explanations for predictive results. This opacity poses challenges in understanding the rationale behind the predictions for specific samples.

Future research should seek to address the current limitations and explore potential avenues for improvement in patient-physician matching systems. A crucial objective is the development of sophisticated algorithms that enhance both the precision and responsiveness of this matching process. The incorporation of real-time data in conjunction with advanced machine learning models may facilitate the dynamic allocation of consultations based on physician availability, which could potentially reduce wait times and enhance patient satisfaction. Moreover, future studies should investigate the integration of multimodal data sources, including patient histories, imaging data, and real-time physiological signals. Such integration could facilitate a more comprehensive understanding of patient conditions, thereby improving diagnostic accuracy and treatment recommendations. Additionally, there is a need to develop explainable models that not only provide accurate recommendations but also offer transparent justifications for their decisions. This transparency would facilitate more informed decision-making in clinical settings, enhancing trust and effectiveness in healthcare.

## 5 Conclusion

This paper presents the RAGPR model, which is designed to improve the performance of triage in web-based medical services. The primary function of this model is to efficiently filter and select appropriate physicians, thereby assisting patients in identifying medical professionals best suited to address their specific healthcare needs. The implementation of this method has significant practical implications, suggesting its potential integration into various healthcare website systems to enhance the quality of physician recommendations.

## Data Availability

The raw data supporting the conclusions of this article will be made available by the authors, without undue reservation.
